# Targeted Gene Delivery through the Respiratory System: Rationale for Intratracheal Gene Transfer

**DOI:** 10.3390/jcdd6010008

**Published:** 2019-02-15

**Authors:** Michael G. Katz, Anthony S. Fargnoli, Sarah M. Gubara, Kenneth Fish, Thomas Weber, Charles R. Bridges, Roger J. Hajjar, Kiyotake Ishikawa

**Affiliations:** Cardiovascular Research Center, Department of Cardiology, Icahn School of Medicine at Mount Sinai, New York, NY 10029, USA; anthony.fargnoli@mssm.edu (A.S.F.); sarah.gubara@mssm.edu (S.M.G.); kenneth.fish@mssm.edu (K.F.); thomas.weber@mssm.edu (T.W.); charles.bridges@mssm.edu (C.R.B.); roger.hajjar@mssm.edu (R.J.H.); kiyotake.ishikawa@mssm.edu (K.I.)

**Keywords:** gene therapy, lung cellular structure, biological barriers, viral vectors, endotracheal route of delivery, hereditary, mutation

## Abstract

Advances in DNA- and RNA-based technologies have made gene therapy suitable for many lung diseases, especially those that are hereditary. The main objective of gene therapy is to deliver an adequate amount of gene construct to the intended target cell, achieve stable transduction in target cells, and to produce a clinically therapeutic effect. This review focuses on the cellular organization in the normal lung and how gene therapy targets the specific cell types that are affected by pulmonary disorders caused by genetic mutations. Furthermore, it examines the pulmonary barriers that can compromise the absorption and transduction of viral vectors and genetic agents by the lung. Finally, it discusses the advantages and limitations of direct intra-tracheal gene delivery with different viral vectors in small and large animal models and in clinical trials.

## 1. Introduction

There are no current conventional pharmacological treatments that can act effectively on intracellular targets. Instead, intracellular targets must be regulated by genes. Effective transcription and translation of DNA into a protein product can change cell behavior to achieve a successful therapeutic outcome. Therefore, gene-based therapy holds promise as a new, effective treatment. The main objective of gene therapy is to deliver an adequate amount of gene construct to the intended target cell, achieve a stable transduction of a target cells, and to produce a clinically therapeutic effect.

Several pulmonary diseases have dysregulated factors that can be therapeutic targets. Thus, gene therapy approaches that can be well suited for hereditary lung diseases with monogenetic mutations are especially attractive given that the correction of autosomal recessive or dominant disorders could be resolved through the exogenous delivery of the wild-type gene. Furthermore, it is a viable approach to modulate disease-causing factors or to help upregulate the factors downregulated in the disease state.

Over the last few decades, much progress has been made in the gene delivery approach to design vectors that achieve specific cell transfer, minimize innate and adaptive immune responses, create new candidates for therapeutic genes, and determine the best route of administration to introduce these genes. To achieve significant pulmonary gene transfection, various vectors have been used so far. These vectors can be classified into three main categories: non-viral, recombinant viral, and engineered hybrid. Compared to other vectors, viruses have an evolutionary advantage in their interactions with the cellular receptors and intracellular trafficking to the nucleus. In this review, we will focus on using only viral vectors for intratracheal gene transfer. The ideal viral vector should be trophic to the lung tissue, result in long-term expression, minimize the risk of innate and adaptive immune response, and possess an enough coding capacity to incorporate the gene and enhancing promoters [1]. Today, Adeno-associated virus (AAV)-based vectors are undoubtedly among the most promising DNA delivery vehicles [2]. Advantages of AAV as a respiratory gene delivery vector include: efficient transduction, minimal cellular immune response and persistent expression of therapeutic gene of interest. Although the technology is advancing rapidly, there are several difficulties associated with AAV. Barriers to integration, re-administration, and the viral vector itself all affect the efficacy of successful gene therapy. A great knowledge base is currently accumulating addressing the challenges associated with this technology. Another interesting application of AAV vector is CRISPR/Cas9 delivery to program genetic and epigenetic manipulations [3,4]. The CRISPR/Cas9 genome-editing system has been rapidly developed and is widely used in all human stem cell studies. In recent years, this system has changed the field of translational stem cell and gene therapy research. Although solutions are still required to reduce the off-target effect, improve editing efficiency, and exploit novel delivery strategies at a low cost. Moreover, implementation of this technology requires safe and effective delivery of all of these components into the nuclei of the target lung tissue [5].

A substantial limitation of AAV vectors is their small packaging capacity that is generally considered to be 4100–4900 bp. This limitation makes vector design for some of the lung diseases challenging. For example, cystic fibrosis trans-membrane conductance regulator (CFTR) gene has a cDNA of 4450 bp, therefore, the application of AAV viral vector in CFTR is limited. To address this problem, strategies based on trans-splicing and homologous recombination were developed [6]. Some studies have successfully used short sequences of endogenous AAV promoters [7]. Other studies have produced AAV vectors with a CFTR cDNA with a deletion at the N terminal, the regulatory domain or missing other transmembrane domains of CFTR These deletions did not alter the function of the CFTR gene [8].

## 2. Cellular Organization in the Normal Lung

The development of gene-based therapeutics for pulmonary application requires a detailed knowledge of the lung’s cell structure. The lungs are paired organs of the respiratory system and can be divided into two major components: the conducting airways, consisting of the trachea, bronchi and bronchioles to transport air from upper respiratory tract to the peripheral lung, and the parenchyma, including alveoli, where gas exchange takes place. The upper respiratory system includes the nasal cavity, paranasal sinuses, and pharynx, while the larynx, trachea, bronchi and bronchioles comprise the lower respiratory tract. The nose and paranasal sinuses act as a first line of defense against inhaled particles through the filtration function and irregular structure of the nasal bones and mucosa. The trachea divides into the right and left main stem bronchi. Each of these give rise to lobes and segmental bronchi. The bronchi branch progressively branches into the bronchioles. The bronchioles terminate in the acinus, which is with capillary net involved in gas exchange and contains alveolar ducts, alveolar sacs, and the alveoli. 

There are about 40 types of cells in the respiratory tract [9]. The epithelium is most important functional cell compartment of the lung and includes airway epithelial cells lining the large, small and terminal airways (trachea, bronchi, bronchioles, and terminal bronchioles/alveolar ducts) and submucosal glands under the airways (basal, club, goblet, ciliated and neuroendocrine cells). The alveolar epithelium (alveolar Type I and Type II epithelial cells) lines alveolar units, and has many essential functions including protective clearance along the mucociliary line and secretion of mucins, growth factors and degradative enzymes [10]. The inner surface of the alveolus is covered by surfactant, which are phospholipids synthesized mostly by type II epithelial cells. In the normal lung, 95% of the alveolar lining is covered by type I epithelial cells. Type II pneumocytes make up 60–65% of alveolar cells. These cells regulate the fluid balance across the alveoli-capillary membrane and secretion of various cytokines. Gas exchange in the lung occurs within alveoli, which are air-filled sacs composed of type II and type I epithelial cells, capillaries, and various resident mesenchymal cells. Interestingly, alveolar type II cells are the main stem cells/progenitors that regenerate during normal homeostasis and injury. Genetic lineage-tracing experiments showed that surfactant protein C–positive type II cells could renew and differentiate over about a year [11]. In addition, epithelium of the mouse trachea and human airways contains a population of basal cells that can function as progenitor cells during postnatal growth and in the adult at steady state [12].

The other main cell functional compartment of the lung is comprised of pulmonary vascular endothelial cells, which form micro capillary bed that surround the alveoli where gas-exchange occurs. The lung also contains supporting cells such as: (i) smooth muscle cells lining both airways and vascular structures; (ii) supporting fibroblasts in the alveoli units; (iii) hematopoietic and immune cells; and (iv) neuronal cells of the pulmonary nerves. Many of these supporting cells make up part of the interstitial space, which is located between the alveolar epithelium and capillary endothelium membrane. The interstitium is formed by an extracellular matrix and contains the cells listed previously as well as alveolar macrophages, capillary pericytes, myofibroblasts, mast cells, lymphocytes and granulocytes.

## 3. Target Cells for Gene Therapy in Different Lung Diseases with Genetic Backgrounds

Currently, genetic etiologies have been identified for several lung diseases. With advances in nucleic acids research and engineering, gene transfer targeting specific cells is an emerging technology.

Lungs are highly permeable and have large, absorptive surface areas in the 70–140 m^2^ range with extremely thin absorptive mucosal membranes and receive multiple arterial blood supplies from the bronchial and pulmonary arterial systems [13]. The respiratory epithelial cells play prominent roles including regulation of airway tone, production of airway lining fluid, and absorption of most known macromolecules [14,15]. With these properties, the potential for gene delivery via the airway becomes a very important and promising therapeutic approach. Topical delivery of medications via pulmonary route has been already documented for some respiratory diseases including asthma, pulmonary hypertension, pneumonia, bronchitis etc [16,17]. Moreover, many other drugs, which are currently injected intravenously, such as growth hormones, glucagon, or insulin, could possibly be delivered by local airway route because the efficiency of this method is better.

Depending on the underlying disease, the target cells in the lung can vary from epithelial cells, alveolar cells, macrophages, smooth muscle or endothelial cells. Below we will review several genetic lung diseases and the cell types involved in their pathogenesis.

### 3.1. Pulmonary Arterial Hypertension

Pulmonary arterial hypertension (PAH) is a devastating disease characterized by abnormal proliferation of pulmonary vascular endothelial and smooth muscle cells. The disease causes progressive dyspnea and right heart failure. A heterozygous mutation in the *bone morphogenetic protein receptor type-II* (*BMPR-II)* gene, a member of the transforming growth factor superfamily of receptors was documented in ~70% of patients with hereditary PAH and ~20% of patients with idiopathic PAH. The possibility of treating PAH via modulating dysregulated *BMPR-II* signaling is thus a rational consideration. Targeted delivery of adenoviral vector containing the *BMPR II* gene to pulmonary vascular endothelium in rodent PAH models resulted in substantially reduced severity of PAH, providing evidence for the rationale for gene therapy in the setting [18]. The cells most involved in the etiology of PAH are vascular endothelial cells, vascular smooth muscle cells, and lung fibroblasts.

### 3.2. Cystic Fibrosis

Cystic fibrosis (CF) is an autosomal recessive hereditary disorder resulting from mutations in the *cystic fibrosis trans-membrane conductance regulator (CFTR)* gene. The *CFTR* gene regulates chloride and sodium ions and the balance of electrolytes in the respiratory system across epithelial membranes. Thus, this gene has an important role in a number of cell functions. With a broad spectrum of effects, it is unlikely that one pharmacologic drug would be capable of restoring the multiple regulatory functions of *CFTR*. Mutations of this gene result in different effects as a function of pulmonary cell type. CFTR is located in the apical membrane of the airway epithelial cells. There is extensive evidence that the pulmonary abnormalities in CF are initiated by a deficiency of CFTR function in the epithelium. Therefore, the primary target for gene therapy of cystic fibrosis is the epithelium of the lower respiratory tract, which is mostly contained in the well-differentiated ciliated columnar airway epithelial cell. In addition, it was reported that mesenchymal stem cells (MSC) are an attractive therapeutic in murine models of CF. MSCs were shown to improve lung morphology, decrease neutrophil recruitment into the lung, and promote the resolution of infection in CF lungs [19]. Probably, both endogenous and exogenous stem and progenitor cells can stimulate lung regeneration and repair in cystic fibrosis. These cells are believed to maintain tissue homeostasis and become activated to replenish damaged tissue.

The strategy to repair the mutated *CFTR* by delivering a normal *CFTR* gene to the respiratory epithelium is very attractive for a number of reasons. Cystic fibrosis is a recessive genetic disease, so the cellular defect can be corrected by a single normal copy of the gene. Furthermore, the respiratory epithelium, may be accessible by application of vector/gene construct within the airway, allowing for directed introduction of reparative genes to the primary target organ [20]. With the demonstration that the normal *CFTR* cDNA could be transferred and expressed in the airway epithelium in vivo, it is logical to hypothesize that this could be a prospective approach to managing this disease [21].

### 3.3. α-1 Antitrypsin Deficiency

Alpha-1 antitrypsin (A1AT) is an enzyme produced by the liver that inhibits the action of proteases and protects the lungs from the damaging effects caused by the activation of neutrophil elastases and other proteinases such as proteinase-3, cathepsins, and metalloproteinases. A1AT deficiency is a genetic disorder and results from an inherited mutation in the gene that controls the production and release of the enzyme. The disorder results in a protease/antiprotease imbalance, causing interstitial lung damage and destruction of alveolar wall. Destruction of the alveolar wall severely inhibits gas exchange in the alveolar capillary membrane and causes excessive cell death of structural cells comprising the alveolus, leading to lung inflammation and emphysema [21,22]. It was demonstrated intracellular uptake of A1AT in the pulmonary endothelial cells and smooth muscle cells occurs in a time- and dose-dependent manner. A1AT internalization is actively regulated via endocytosis. This effect of A1AT is closely connected to the lung’s vascular endothelial and smooth muscle cells [23].

### 3.4. Surfactant Protein-B Deficiency

Surfactant Protein B (SP-B) deficiency is a congenital lung disorder characterized by severe respiratory insufficiency and is unresponsive to any treatment except lung transplantation. SP-B deficiency is an example of inherited disease which is the result of a mutation in a single gene [24]. Replacement of SP-B with commercially available surfactant proteins provides only transient improvement in lung function and is largely ineffective. All known pathophysiological mechanisms of this disease lead to pulmonary epithelial cell and type II pneumocytes cell dysfunction. For gene therapy to be an effective treatment for SP-B deficiency, the gene encoding the protein should be delivered to the nucleus of type II pneumocytes [25]. The current pharmaceutical management of SP-B deficiency involves repeated administration of artificial surfactant. With gene therapy, the level of surfactant expression can be controlled on the intracellular level thus eliminating the need for repeated administration.

## 4. Pulmonary Barriers to Gene Transfer

**Figure 1:**
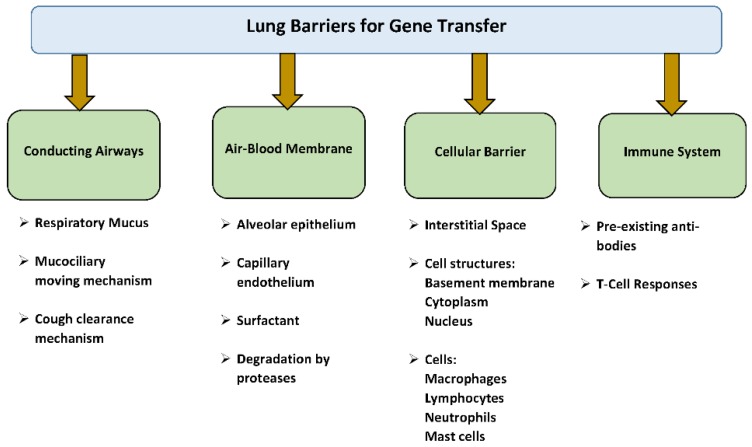
Lung barriers for gene transfer.

In general, there are several biological barriers in the lungs that make absorption of foreign materials difficult. These barriers can be divided into: the conducting airway barriers, air–blood membrane (alveolo–capillary) barriers, cellular barriers, and the immune system [26] (Figure 1). The conducting airway barriers include respiratory mucus, mucociliary and cough clearance mechanism, alveolar lining layer, alveolar epithelium, basement membrane, enzymes created by pulmonary tissue, macrophages and several other cells like lymphocytes, neutrophils and mast cells. These respiratory barriers can compromise the absorption of viral vectors and genetic agents by the lung, proposing a problem for gene therapy.

### 4.1. Respiratory Mucus, Mucociliary, and Cough Clearance Mechanism

The mucus layer lays on top of the cilia in the nasal airway, pulmonary airway, and trachea. It consists of glycosylated macromolecules. Respiratory mucin containing secretions by Goblet cells and submucosal glands contribute to the formation of a mucus layer. The inhaled vectors are captured in the mucus via three mechanisms: electrostatic interactions, hydrophobic forces, and hydrogen bonding. They are cleared from the lung via mucociliary defense clearance by moving gene particles to the pharynx. An additional mechanism to clear mucus is cough clearance. Interestingly, lung infection and inflammation enhance this barrier [27,28]. The low airways at the level of the bronchioles are lined by ciliated airway epithelial cells which act as an additional barrier. It was shown that use of viscoelastic gel formulations can inhibit mucociliary clearance and increase viral gene transfer to the rodents’ airways [29]. 

### 4.2. Air–Blood Barrier

Pulmonary gene absorption is largely determined by the epithelial cells and their basement membrane, and the lung’s interstitial space containing a variety of cells, collagen, elastic fibers, interstitial fluid and lymphatic vessels. Absorption across the alveolar epithelium includes several mechanisms like passive and active movement through the cells and intracellular pores, vesicular transport, and drainage through the lymphatic’s vessels. Drugs are absorbed from the alveolo-capillary membrane into the blood must via the surfactant layer, the lining fluid, the epithelium, its basement membrane, the interstitium and the endothelium. The last layer, the alveolar–capillary endothelium, is extremely thin and has a relatively large number of endocytic vesicles and is not considered the major barrier to vector transport. 

The alveolar epithelium lines the alveoli with their cytoplasm, basal lamina, and capillary endothelium. On reaching the alveoli, many vectors can be degraded by proteases or removed by alveolar macrophages. The alveolar epithelium is also covered with a layer of pulmonary surfactant, which comprises phospholipids and specific surfactant-associated proteins. It is an active defense complex, which is synthesized and secreted primarily by type II alveolar pneumocytes. The major function of surfactant is to reduce surface tension at the air–liquid interface of the terminal airways, thereby reducing the tendency of alveoli to collapse [30]. However, the components of surfactant like lipids and proteins may create a potential barrier for gene delivery to epithelial cells [31]. Lung surfactant may induce aggregation and, thus, potentially compromise entrapment virus to alveoli.

### 4.3. Cellular and Immune Barriers

Alveolar macrophages are the most numerous cell type in the alveolar space and are capable of suppressing the alveolar inflammation and inducing an adaptive immune response through antigen presentation to T-cells. The pulmonary macrophages secrete peroxidases, and immunomodulatory mediators like interleukins, leukotrienes and proteases as part of the host defense mechanism. They are long-lived cells with a relatively slow turnover rate of ∼40% per year. It has been shown that they participate in the resolution of inflammation by clearing cellular debris, apoptotic neutrophils, and recruiting anti-inflammatory monocyte-derived macrophages. Displaying a unique phenotype, these cells maintain the lung’s homeostasis and help remodel the lung parenchyma after tissue injury and infection [32]. Alveolar macrophages inhibit virus-mediated gene transfer to pulmonary epithelia, and the mechanism of inhibition involves uptake of the viral particles into macrophages [33]. After gene transfer internalization of the virus, expression of inflammatory mediators is initiated within alveolar macrophages [34]. The immune system can also affect viral gene transfer. The presence of pre-existing antiviral antibodies due to prior infection can neutralize virus infectivity with no therapeutic response. The inherent immunogenicity associated with viral vectors include:

1. Pre-existing antibodies and T- cell response against viral vectors
2. Neutralizing antibodies generated against capsid proteins of viral vector
3. T-cell-mediated response against viral vector capsid
4. Neutralizing antibodies and T cell-mediated response against transgenes

Apart from pre-existing antibodies, cellular and humoral immune responses can develop after administration of the viral vector, which may render subsequent virus injection much less effective. 

The epithelial portion of the lung is another barrier for gene transfer into the lung due to the low efficiency of endocytosis across the apical membrane and tight junctions between cells that disturb the uptake of gene vectors into the baso-lateral surface [35,36]. This is an additional challenge for adenovirus and lentivirus-mediated gene therapy since their receptors are mostly localized on the baso-lateral membrane of airway epithelium [37].

## 5. Rationale for Intratracheal Gene Delivery to the Lung

**Figure 2:**
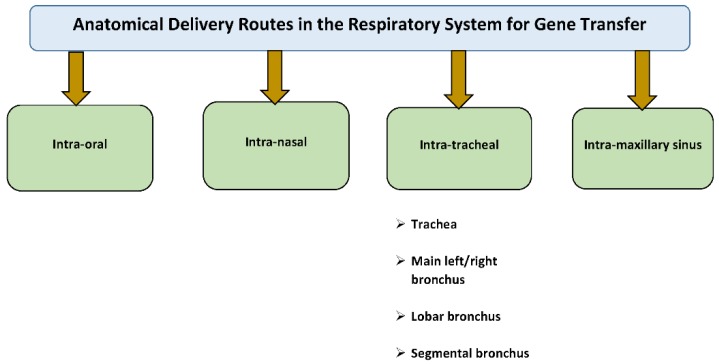
Anatomical delivery routes in the respiratory system for gene transfer.

Pulmonary gene transfer has become an attractive therapeutic approach, as there is a clinical need for long-term pharmaceutical interventions to address both inherited and acquired pulmonary diseases. Compared to other organs, the lung has a unique surface area for deposition and uptake of proteins including different vectors with gene constructs either for local or systemic aims. Through the pulmonary airway routes, genes can be administered intranasally or orally using inhalation devices, intratracheal instillation, and intratracheal inhalation with and without a bronchoscope. Intravascular pulmonary artery injection is an additional but invasive option (Figure 2).

**Figure 3:**
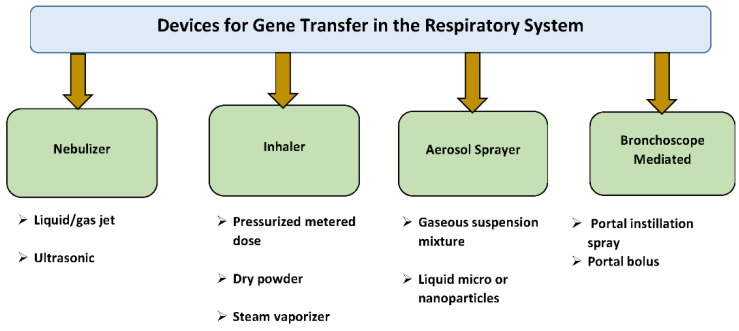
Devices for gene transfer in the respiratory system.

Direct airway delivery to the lungs is a very desirable and noninvasive route of gene administration. Compared to intravascular delivery, airway delivery has much lower endonuclease activities that can destroy DNA and RNA molecules [38]. Direct gene application to the pulmonary tissue can minimize systemic adverse effects while avoiding liver first-pass metabolism and hepatic absorption [38] (Figure 3).

Flexible bronchoscopy (FB) is a minimally invasive procedure that is widely used in humans and large animals, like sheep and pigs, for intratracheal delivery. FB allows the investigator to perform endoscopic examination of the tracheobronchial tree and choose the anatomical structure where the gene will be administered. In humans, the trachea is a continuation of the larynx that extends from the lower border of the cricoid cartilage to the carina. At the carina, the trachea bifurcates into the right and left main stem bronchi. The right main stem bronchus branches off at an angle of about 25 degrees with respect to the trachea. The right upper lobe bronchus trifurcates into the apical, posterior, and anterior divisions. The right main stem bronchus continues as the bronchus intermedius, which gives rise to the middle lobe bronchus and lower lobe bronchus. The middle lobe bronchus bifurcates into the medial and lateral branches. Segmental bronchi from the right lower lobe bronchus include the superior, anterior basal, medial basal, lateral basal and posterior basal divisions. The left main stem bronchus divides into the upper lobe bronchus and lower lobe bronchus. The left upper lobe bronchus has a superior division and an inferior division (also referred to as the lingular bronchus) that supply the upper lobe and lingula, respectively. The superior division bifurcates into the apicoposterior segmental bronchus and anterior segmental bronchus. The lingular bronchus bifurcates into superior and inferior lingular segmental bronchi. The lower lobe bronchus consists of superior, lateral basal, anteromedial basal, and posterior basal segmental bronchi.

In rodents, unlike in humans, the right lung consists of four lobes: cranial, middle, caudal and accessory lobes, and the left lung has only one lobe. The rodent’s trachea divides into the left and right primary bronchi. The right primary bronchus divides into bronchi, which conform to the lobes of the right lung, namely the anterior (apical or cranial), middle (cardiac), median (azygous or accessory) and posterior (caudal) lobes. The left primary bronchus supplies the left lobe. Each primary bronchus gives rise to smaller secondary bronchi from which the bronchioles originate. The rodents lack respiratory bronchioles, characteristic of primate lungs including those of humans. Several techniques are used for the tracheal access in rodents including oro-tracheal intubation, tracheal instillation and tracheotomy.

**Figure 4:**
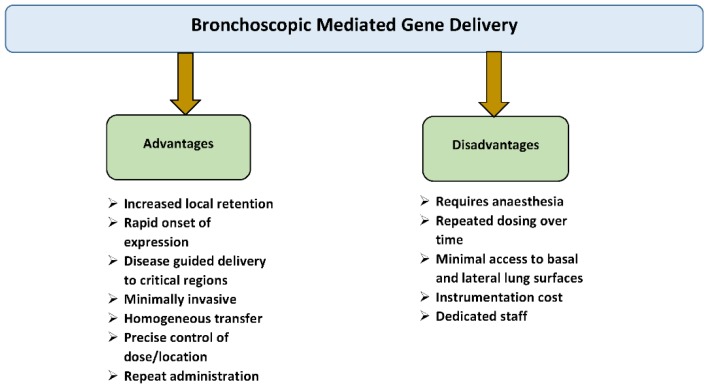
The advantages and disadvantages of bronchoscopic (intratracheal)-mediated gene delivery.

Clinically relevant airway administration has some advantages and limitations. Advantages include: (i) Rapid onset of action, (ii) High local gene concentration, (iii) Delivery directly to the target lung tissue, (iv) The gene stays in the lung instead of being metabolized and excreted, and (v) Needle and pain-free administration [39]. Disadvantages include: (1) The therapy will likely require repeat dosing because all protected mechanisms can destroy the vector/gene construct before it can be achieved by the targeted cells; (2) Delivery is usually limited due to liquid formulations as there is no dry powder or metered-dose formulation currently available for any vector-gene combination; (3) Nebulizer devices do not provide good delivery efficiency; (4) Delivery of the vector to the lungs of patients with chronic lung diseases is non-homogeneous due the fact that vector depositing only in regions of the lungs with good ventilation that could make treatment much less efficacious; (5) The mucus barrier is thick and viscous in the patients with many obstructive and genetic lung diseases; and (6) Many viral vectors recognize their receptors only on the basal or lateral surfaces of the lungs, which are difficult to access [39] (Figure 4). 

The deposition of genes by administration in the pulmonary airway mainly takes place by three mechanisms: gravitational sedimentation, inertial impaction, and diffusion. If the particle size is comparatively bigger than a nanoparticle, the deposition takes place by first two mechanisms. When the particle size is smaller, the diffusion mechanism turns on [40].

The most common and successful method used in laboratory animals is the intratracheal instillation. In this method, a gene solution or dispersion is delivered into the lungs by a special syringe with a microsprayer tip. This provides a fast and quantifiable method of gene transfer. Thus, the instillation process is simple and has uniform drug distribution. In preclinical animal studies, intratracheal instillation has frequently been used to assess pulmonary uptake and systemic bio-distribution, especially with regard to the precise dosing and effectiveness associated with this method. However, intratracheal instillation for application in humans requires endotracheal intubation and anesthesia thus making it difficult to use in clinic. 

## 6. Intratracheal Delivery with Different Viral Vectors in Small Animals and Clinical Trials

### 6.1. Adenovirus

Adenovirus vectors are the most frequently used viral vectors due to the high levels of transgene expression that can be obtained in a many host cells and can be effectively delivered intratracheally. Current versions of adenovirus exhibit reduced toxicity and decreased immune response. The vectors are predominantly nonintegrating, episomal, nonenveloped, double-stranded DNA viruses. In models of bacterial sepsis, a cDNA encoding CTP:phosphocholine cytidylyltransferase (CCTα) enzyme was delivered intratracheally in mice using a replication-deficient adenovirus 5. Gene transfer produced high-level gene expression, increased alveolar surfactant (DPPC) levels and improved lung surface tension and pressure–volume relationships relative to control mice [41]. Repetitive intratracheal instillation of adenovirus 5 vector carrying the *LacZ* *β-galactosidase* gene stimulated a localized and systemic antibody response to the vector [42]. Adenoviral vector containing recombinant human CF transmembrane conductance regulator gene was delivered by bronchoscope in patients with CF. Detectable gene transfer was observed in harvested bronchial epithelial cells [20]. Endobronchial spray of Ad/*CFTR* to patients with CF demonstrated that Ad vector can deliver sufficient levels of CFTR cDNA to the airway epithelium and can protect the lungs from the respiratory manifestations of CF. However, expression was transient, lasting less than 30 days and was achieved with a second administration [21]. Intratracheal lobar administration of Ad/*CFTR* showed that infected cells with the vector included mononuclear inflammatory cells, cuboidal and columnar epithelial cells [43]. To inhibit lung matrix metalloproteinase in rats exposed to monocrotaline, intratracheal instillation of the adenovirus-mediated *TIMP-1* gene was administered. Rats treated with the gene had less severe pulmonary vascular remodeling evidenced by a decreased right ventricular hypertrophy, decreased masculinization of peripheral pulmonary arteries and increased lung-cell apoptosis compared to controls [44]. 

Quantitative reverse transcriptase-polymerase chain reaction (qRT-PCR) demonstrated robust and similar expression of *BMPR-II* gene in all major lung lobes after intratracheal nebulized gene therapy with adenoviral *BMPR-II* delivery [45]. Treatment of idiopathic pulmonary fibrosis (IPF) rats with adenoviral delivery of the *vascular endothelial growth factor (VEGF)* gene resulted in reduced endothelial apoptosis, increased vascularization, and improved PAP due to reduced remodeling but worsened PF [46]. The *Kv1.5* gene is an important O_2_-sensitive, voltage gated K^+^ channel and potential therapeutic target in PH. Administration of *Kv1.5* to the pulmonary circulation via an intratracheal aerosol is feasible and effective in eliciting transgene expression in PA smooth muscle cells (SMCs). This treatment reduces pulmonary vascular resistance (PVR) and restores hypoxic pulmonary vasoconstriction (HPV) in rats with established CH-PHT [47]. 

### 6.2. Adeno-Associated Virus

Adeno-associated viruses are becoming increasingly investigated as gene delivery vectors due to a lack of pathogenicity, low immunogenicity in humans, lack of integration into the host genome, ability to infect dividing and non-dividing cells of many tissues, and prolonged transgene expression. However, studies have also revealed challenges of AAV’s as gene delivery vectors, including species specific variable transfection rates, low organ targeting efficiencies, and neutralization by pre-existing circulating antibodies. 

Intratracheal delivery of rAAV5 and rAAV1 vectors resulted in transduction of lung Clara cells and alveolar type II cells that proliferated following lung injury. Both of these AAV vectors appeared to preferentially transduce conducting airway epithelial progenitors with the capacity to clonally expand, both in vivo following lung injury and in vitro [48]. AAV2/9 vectors can penetrate the epithelial layer and have the potential to transfect target cells deep into the lung tissue. A gene expression of AAV2/9-mediated human *A1AT* gene expression in serum was ≈60-fold better than that of AAV2/5. The AAV2/9-mediated *LacZ* *β-galactosidase* gene transfer in lung airways was stable for 9 months, suggesting that a progenitor airway cell population was transduced [49]. Studies with endotracheal administration of AAV1 and AAV5 in the rat lung showed strong marker gene expression in the airway epithelial cells and confirm their advantages over AAV2 via the same delivery method. AAV was well tolerated in vivo, without any significant impact to lung growth. However, transgene expression remained lower than that obtained with an adenoviral vector [50]. Viral vectors derived from AAV2/6 with tropism for the airway epithelium were injected into the airway of fetal mouse. Favorable rates of transfection were reported for direct injection of rAAV2/6.2 in the fetal mouse airway over other methods. Histological analysis for β-galactosidase revealed 17.5% of epithelial cells transduced in the conducting airways compared to only 1.5% in the alveolar cells. Stable gene expression was observed up to one month after injection [51]. Another study demonstrated that AAV2/5 transduces both the upper and lower mouse airways and that transduced cells remain present until 15 months. Using cell-specific markers, transduced cell types were identified as being ciliated, Clara, and alveolar type II cells. As well, this vector was successfully re-administered 14 months after primary vector administration, resulting in increased gene expression in the lung [52]. 

## 7. Intratracheal Delivery with Different Viral Vectors in Large Animals

Large animal models offer testing of gene therapy approaches in more clinically relevant conditions in terms of lung size, vector dose, and deliver method. A huge advance in CF gene therapy was the recent generation of a pig CF model [53]. This model offers preclinical CF gene therapy studies in a model that has similarities in airway anatomy and chloride channel compositions compared to humans [54,55]. In contrast to CF, there are few large animal models of pulmonary hypertension. Methods that are used to induce pulmonary hypertension in rodents are either less reliable or technically challenging. Large animal models that have been proposed include pulmonary vein banding, aorta-venous shunt, and pulmonary embolism [56,57,58]. Pulmonary fibrosis may be induced by injection of bleomycin and there are species that develop spontaneous pulmonary fibrosis [59,60]. Other genetically induced models may soon become available using rapidly advancing gene editing technologies.

### 7.1. Adenovirus

Adenoviral gene therapy for CF advanced rapidly into clinical trial after demonstration of effective expression in rats [61,62]. Around the same time the first patients were administered, Bout et al. demonstrated successful transgene expression using adenovirus through bronchoscope in non-human primates [63]. However, the transgene expression using adenovirus was short and with the emergence of AAV, which has more long-term expression and better safety profiles, the fields interest shifted to AAV.

### 7.2. Adeno-Associated Virus

As the limitation of adenovirus for long-term expression became aware in clinical trial, CFTR gene therapy was tested using AAV2 in rabbits and then macaques [64,65]. These studies showed long-term expression without apparent concerns on safety and prompted clinical testing [66]. Although the Phase I clinical trial demonstrated potential efficacy with good safety profile, subsequent Phase II study failed to meet the efficacy endpoint [67]. The results of this trial despite a good safety led to a search of more effective AAV serotypes for airway gene therapy.

In a recent study, bronchoscopy delivery of different AAV serotypes encoding GFP in 1 week-old sheep resulted in transgene expression of small and medium sized airways with AAV1, AAV6, AAV8, AAV9, and AAVrh10, but not AAV5 [68]. In contrast, when recombinant AAV5 was aerosolized into Rhesus macaques, AAV5 was able to express both GFP and truncated form of CFTR in airway cells [69]. Recently, Steines et al developed a variant of AAV using directed evolution approach and aerosolized it into pig airway [70]. They demonstrated efficient transduction of CFTR in pig airway epithelia together with improvement in airway surface PH and bacterial-killing function [70]. Although the study confirmed functional improvement after airway *CFTR* gene therapy in clinically relevant condition, their chimeric AAV failed to transduce human airway epithelia in vitro. Another in vitro study also showed that airway epithelia from human and Indian Rhesus monkeys exhibited significant differences in gene transduction efficiency [71]. These results indicate challenges in finding the most effective AAV serotype for expressing genes in human epithelia.

One emerging avenue of airway gene therapy is the targeting of vascular cells for treating pulmonary hypertension using vascular tropic AAV serotype. Following the demonstration of pulmonary vascular remodeling inhibition with airway AAV1.*SERCA2a* gene delivery in a rodent model, this program was advanced to a large animal experiments [72]. Using a pig model of pulmonary hypertension induced by pulmonary vein banding, it was reported that airway AAV1.*SERCA2a* gene delivery with use a bronchoscopy sprayer and nebulization approach prevents progression of vascular remodeling and development of pulmonary hypertension [73]. No safety issues were identified after using AAV gene therapy in pulmonary application.

## 8. Re-Administration of AAV Vectors for Lung Gene Transfer

For the majority of clinical applications that aim to deliver therapeutic genes to the lung with AAV vectors, long-term expression of the therapeutic proteins will be essential. Due to the turnover of lung epithelial and other target cells in the lung, the over-expression of delivered genes will decline with time. Therefore, infrequent vector re-administration will be necessary for pulmonary epithelial targets. The reason for this is that AAV genomes largely persists episomally, and cell division of the pulmonary cells will eventually result in a progressive reduction in vector genomes per cell and a concomitant decrease in expression of the therapeutic protein. Systemic re-administration of recombinant AAVs is recognized as challenge and remains an unmet need for systemic re-administration. The potential for repeated administration to the lung, however, has not been established yet.

Data obtained from preclinical animal experiments is complex. For instance, Miller and colleagues reported that, in mice, administration of recombinant AAV serotype 2 expressing *LacZ β-galactosidase* essentially blocks subsequent transduction by an AAV2 vector expressing alkaline phosphatase [74]. In contrast, administration of AAV2 encoding *LacZ β-galactosidase* followed by the delivery of AAV6 encoding alkaline phosphatase only modestly reduced transduction by the latter vector. These data suggest that administration of a different serotype might be a promising approach for re-administration of viral vectors [74]. Delivery of AAV6 expressing alkaline phosphatase in mice after delivery of AAV6 encoding LacZ resulted in significant alkaline phosphatase expression [74]. Taken together, these results suggest that AAV6 is less immunogenic compared to AAV2, at least in mice, a hypothesis supported by the levels of neutralizing antibodies in sera immunized with either AAV 2 or AAV6 [74].

The same group reported earlier that even if the transgene is delivered with a AAV serotype 2 vector, re-administration is possible if the immune system is transiently suppressed by the co-administration of an antibody against CD40 and a fusion protein of an IgG constant region with the extracellular domain of CTLA4. The former inhibits a B-cell antibody response, whereas the latter reduces T-cell activation by binding to the CTLA4 receptor CD86 [75,76,77]. 

Wilson et al. group demonstrated that when an AAV5 vector encoding erythropoietin is delivered by intranasal instillation expression of erythropoietin progressively decreased over a time span of 5 months. Strikingly, however, intranasal administration of an AAV5 vector encoding β-galactosidase to the mice that were previously injected with AAV5. erythropoietin resulted in near identical expression levels of β-galactosidase when compared to naïve mice [78]. Given the fact that the serum neutralizing antibody titers were between 1:160 and 1:320, these results are truly astonishing [78]. Wilson’s groups also reported similar results with AAV9 [49]. Conversely, when Hyde and colleagues administered AAV5 vectors (with both an AAV5 capsid and AAV5 ITRs) encoding luciferase to mice that had previously been injected with AAV5 that encoded a different transgene, luciferase expression was dramatically decreased [79].

To date, very little data is available for the efficiency of repeated administration of aerosolized AAV in humans. To our knowledge, the repeated administration of an AAV2 vector encoding the CF transmembrane regulator to treat CF is the only clinical trial that tested the hypothesis that repeat application of aerosolized AAV can be used to treat lung disorders. Unfortunately, this trial failed to meet its primary endpoint [80].

Clearly, repeated application of aerosolized AAV as a feasible therapeutic modality will need extensive further investigation for further translation to the clinic. Regrettably, the immune system of mice and humans are quite different, and only future clinical trials will ultimately yield definitive answers whether repeat administration of aerosolized AAV is a viable treatment strategy.

Taken together, these studies demonstrate the therapeutic potential of AAV vectors for pulmonary gene transfer in a preclinical setting, suggesting further development of an AAV-based gene therapy for human airway diseases.

For many lung diseases, effective gene therapy will require stable genetic transfection of terminally differentiated cells or of progenitor cells that divide infrequently. Lentiviral vectors can integrate reliably into the chromosomes of non-dividing cells. Lentiviruses are a family of complex retroviruses typically associated with infection of lymphocytes and macrophages. Retroviral vectors, unlike other vectors are highly efficient at integrating into the genome of the target cell and do not transfer any viral gene, thus alleviating the risk of vector-mediated toxicity and immune response against the transduced cells. The results support the hypothesis that after intratracheal delivery, lentiviral vectors can overcome physical and biochemical barriers present on normal airway epithelium and efficiently transduced the proximal and distal surface epithelia, and to a lower extent, alveolar epithelium [81]. It was found that a single dose of lentivirus produced immediate and lifetime mouse airway expression, confirming our hypothesis that use of an integrating vector extends transgene expression. In CF-null mice, functional correction of the CFTR defect can be achieved at least for 12 months [82]. 

## 9. Conclusions

Intratracheal gene delivery is a promising approach to treat both acquired and inherited lung diseases. Compared to systemic and local gene delivery routes including intra-oral and intra-nasal routes, intratracheal instillation/spray with bronchoscope has many benefits including rapid onset of action, achievement of high local gene concentration in targeted cells with minimizing systemic effects and homogeneous distribution. Though there are hurdles to gene therapy that still exist and testing that still needs to be done before treatment options are available in clinic, much progress has been made in the gene therapy approach to design vectors to achieve specific cell transfer, minimize innate and adaptive immune responses, create new genes, and determine the best route of administration. Producing a successful gene therapy treatment for genetic lung diseases may prove useful for treating other genetic diseases in other organs and cells in the body.
